# The Potential of *Metarhizium anisopliae* Blastospores to Control *Aedes aegypti* Larvae in the Field

**DOI:** 10.3390/jof9070759

**Published:** 2023-07-18

**Authors:** Simone Azevedo Gomes, Aline Teixeira Carolino, Thais Berçot Pontes Teodoro, Gerson Adriano Silva, Ricardo de Oliveira Barbosa Bitencourt, Carlos Peres Silva, Abeer M. Alkhaibari, Tariq M. Butt, Richard Ian Samuels

**Affiliations:** 1 Laboratório de Entomologia e Fitopatologia, Universidade Estadual do Norte Fluminense Darcy Ribeiro, Campos dos Goytacazes, Rio de Janeiro 28013-602, Brazil; simoneazgomes@yahoo.com (S.A.G.); carolinoat@gmail.com (A.T.C.); thaisbercot1@gmail.com (T.B.P.T.); silva.gersonadriano@gmail.com (G.A.S.); ricoliver@gmail.com (R.d.O.B.B.); 2 Departamento de Bioquímica, Universidade Federal de Santa Catarina, Florianópolis 88040-900, Brazil; carlos.peres@ufsc.br; 3 Department of Biology, Faculty of Science, University of Tabuk, Tabuk 71491, Saudi Arabia; aalkhaibari@ut.edu.sa; 4 Department of Biosciences, Swansea University, Swansea SA2 8PB, UK; t.butt@swansea.ac.uk

**Keywords:** biological control, entomopathogenic fungi, virulence, persistence

## Abstract

Entomopathogenic fungi are promising as an environmentally benign alternative to chemical pesticides for mosquito control. The current study investigated the virulence of *Metarhizium anisopliae* blastospores against *Aedes aegypti* under both laboratory and field conditions. Virulence bioassays of conidia and blastospores were conducted in the laboratory, while field simulation bioassays were conducted under two conditions: totally shaded (TS) or partially shaded (PS). In the first bioassay (zero h), the larvae were added to the cups shortly after the preparation of the blastospores, and in the subsequent assays, larvae were added to the cups 3, 6, 9, and 12 days later. The survival of the larvae exposed to blastospores in the laboratory was zero on day two, as was the case for the larvae exposed to conidia on the sixth day. Under TS conditions, zero survival was seen on the third day of the bioassay. Under PS conditions, low survival rates were recorded on day 7. For the persistence bioassay under PS conditions, low survival rates were also observed. *Metarhizium anisopliae* blastospores were more virulent to *Ae. aegypti* larvae than conidia in the laboratory. Blastospores remained virulent under field simulation conditions. However, virulence rapidly declined from the third day of field bioassays. Formulating blastospores in vegetable oil could protect these propagules when applied under adverse conditions. This is the first time that blastospores have been tested against mosquito larvae under simulated field conditions, and the current study could be the basis for the development of a new biological control agent.

## 1. Introduction

*Aedes aegypti* (Linnaeus, 1762) vectors dengue (DENV), Zika (ZIKV), and chikungunya (CHIKV) arboviruses, which pose a risk to over half the world’s population, affecting millions of people annually [1]. The extensive use of synthetic insecticides for mosquito control has resulted in insecticide resistance and a corresponding increase in *Aedes*-transmitted diseases [2]. *Aedes* mosquito populations have become resistant to virtually all currently available classes of insecticide [3,4,5,6]. To reduce dependency on synthetic insecticides, research has been carried out to investigate the potential of entomopathogenic fungi (EPF) to control insect vectors [7,8,9,10].

EPF belonging to the genera *Metarhizium* and *Beauveria* are already sold for the control of crop pests and show promise for the control of arthropod disease vectors, including sandflies, kissing bugs, ticks [11,12,13,14], and mosquitoes [15,16,17,18,19,20,21,22]. EPF cause mortality following the ingestion of inoculum (conidia, blastospores) or by the direct penetration of the host cuticle and subsequent colonization of the host body [15,23]. One major advantage of EPF over the extensively used entomopathogenic bacterium, *Bacillus thuringiensis israelensis*, is that it is not restricted to the control of mosquito larvae, but can also kill adults, pupae, and eggs [8,24,25,26,27].

Hydrophilic blastospores and hydrophobic conidia can be produced in liquid media or solid substrates, respectively [27,28]. Conidia production can take 15–21 days whereas blastospores can be produced within 2–4 days [29]. The thin-walled blastospores are marginally more virulent than conidia against the larvae of *Aedes*, *Culex,* and *Anopheles* mosquito species [15,21,30]. The fact that blastospores have multiple routes of entry (cuticle and gut) may explain why these propagules killed *Ae. aegypti* larvae in a relatively short time (12–24 h), significantly quicker than when larvae were exposed to conidia [16,22].

Studies have shown that *M. anisopliae* conidia did not adhere to the mosquito larval cuticle nor germinate within the insect gut, being expelled in fecal pellets [7,31]. It has been suggested that *M. anisopliae* conidia caused stress-induced mortality, a very different mode of action to that normally seen when fungi infect and kill terrestrial hosts [7]. In addition, Daoust and Roberts [32] showed that *M. anisopliae* conidia attached to the respiratory siphon in order to infect *Ae. aegypti*, *Culex pipiens*, and *Anopheles stephensi* larvae. *Metarhizium anisopliae* blastospores were also more virulent than conidia against *Ae. aegypti* pupae and, interestingly, sporulation was observed on the pupae after exposure to blastospores [27].

We also recently demonstrated that blastospores are highly virulent to adult *Ae. aegypti* under laboratory conditions [26], killing mosquitoes more rapidly than conidia; however, virulence declined over time. Previous studies have shown that blastospores are highly virulent under laboratory conditions, but it is also important to test the efficiency of these fungal propagules under conditions similar to the natural situation.

Here, the virulence of *M. anisopliae* conidia and blastospores against *Ae. aegypti* larvae from a native population was first assessed under laboratory conditions. Then, blastospore virulence was investigated under simulated field conditions. Finally, blastospore virulence over time (persistence) was also investigated. This study is a very important step forward in the development of a new biological control agent for use against *Ae. aegypti* larvae.

## 2. Materials and Methods

### 2.1. Mosquito Collection and Rearing

*Aedes aegypti* larvae were reared from eggs collected at strategic sites on the Campus of the State University of North Fluminense (UENF). *Aedes aegypti* populations derived from eggs collected in the field are considered fitter than the more homogeneous, laboratory-reared mosquitoes, and therefore more appropriate for use for investigating pathogen virulence. The eggs were collected using “ovitraps”, which consist of black plastic plant pots (12 cm in diameter × 15 cm in height) with 4 wooden strips or paddles (3 × 12 cm) placed vertically within the pots, providing a highly conducive landing platform for gravid, ovipositing females (See Supplementary Material Figure S1). Approximately 300 mL of tap water was added to each ovitrap before placing them outdoors at sites that were protected from rain and direct sunlight and close to the University Insectary (Latitude: −21°45′8.17″ S; Longitude: −41°19′49.58″ W). After 5 days, the paddles with eggs were collected and dried at room temperature for 24 h. To initiate egg hatching, the paddles were submerged in water and the emergent larvae were maintained in plastic trays (approximately 100 larvae per 100 mL) and fed on freshly ground, autoclaved commercial fish food (Nuvilab, São Paulo, Brazil; 0.05 g per L).

### 2.2. Fungal Isolate

*Metarhizium anisopliae* isolate LEF 2000 was obtained from a soil sample in Campos dos Goytacazes, Brazil (Latitude: −21°45′8.17″ S; Longitude: −41°19′49.58″ W). LEF 2000 is part of the fungal collection of the Insect Pathology Group (UENF). Fungi are maintained on freeze-dried rice grains at −20 °C. This isolate was obtained from a soil sample using an insect bait method, and consecutive purification was carried out on media + antibiotics using standard procedures. Subsequently, monosporic culturing was performed. This isolate has been previously shown by our research group to be highly virulent against *Ae. aegypti* larvae [24], pupae [27], and adults [26] under laboratory conditions.

### 2.3. Conidial Production and Suspensions

*Metarhizium anisopliae* conidia were obtained by culturing the fungus on medium containing potato, dextrose, and agar (PDA), maintained in an incubator for ten days at 27 °C and 70% RH. After this time, conidia were carefully removed from the culture medium with the aid of a spatula, and a suspension was prepared using Tween 80 (0.01%) at a concentration of 1 × 10^7^ conidia mL^−1^, estimated using a Neubauer hemocytometer. Viability tests were carried out by plate counting, and only batches with > 90% germination were used in the experiments.

### 2.4. Blastospore Production and Suspension

Blastospores were produced in corn steep medium liquid consisting of 3% (*v*/*v*) corn steep liquor (Sigma-Aldrich, São Paulo, Brazil), 4% yeast extract (*w*/*v*), and 4% glucose (*w*/*v*). Briefly, 500 µL of a 1 × 10^7^ mL^−1^ conidial suspension was added to 250 mL Erlenmeyer flasks containing 50 mL of culture medium. The flasks were incubated at 27 °C in an orbital shaker at 152 rpm, and blastospores were harvested after three days. The blastospores were separated from the hyphal fragments using a “Miracloth” filter (Sigma-Aldrich, São Paulo, Brazil), and the yield was estimated using a Neubauer hemocytometer. A final concentration of 1 × 10^7^ mL^−1^ was used in the following assays. All batches were tested for viability by plating out suspensions on PDA. Only batches with >90% colony-forming units were used in the experiments.

### 2.5. Laboratory and Semi-field Bioassays

The bioassays were carried out using a fungal concentration of 1 × 10^7^ propagules mL^−1^. In the laboratory, bioassays were conducted to compare the virulence of conidia and blastospores. These bioassays were carried out in a BOD at 25 °C and a photoperiod of 12 h light: 12 h dark. For the field simulation bioassays, blastopore virulence was monitored under totally shaded conditions (TS) or partially shaded conditions (PS) at ambient temperatures (see Section 3). Ten 2nd or 3rd instar larvae (L_2/3_) were transferred to 100 mL cups with 50 mL of fungal suspensions. The larvae from the control groups were exposed to Tween 80 at 0.01% (conidial control) or pure distilled water (blastospores control group). For each bioassay group, four cups were used (10 larvae per cup), totaling 40 larvae. Three replications were performed; therefore, 120 larvae were used in total per treatment. Larval survival was monitored daily for seven days by visual observation. Dead larvae were removed from the cups.

### 2.6. Blastospore Persistence under Laboratory Conditions

Bioassays were conducted to verify how long the blastospore suspensions remained virulent. Recipients were maintained on the laboratory bench at approximately 25 °C, 50% RH, and 12 h light: 12 h dark. *Aedes aegypti* larvae were added to each recipient at different times after the preparation of suspensions as follows: time zero (T0 = the larvae were immediately exposed to blastospores as soon as the suspension had been prepared); 3 days (T3 = the larvae were exposed to blastospores 72 h after preparing the suspension); and larvae were also exposed to blastospores at T6, T9, and T12 days after the preparation of the suspensions.

### 2.7. Blastospore Virulence When Tested against Larvae under Totally Shaded and Partially Shaded Conditions in the Field

Semi-field bioassays were performed using blastospore suspensions at 1 × 10^7^ mL^−1^, and experiments were carried out on two balconies outside the Dengue Research Unit at UENF. One of the balconies, due to its position, did not receive direct sunlight at any time of the day and was denominated as totally shaded (TS) (Supplementary Material Figure S2A). On the other balcony, the experimental area received direct sunlight at certain times of the day (from around 11 am to 4 pm) and was thus denominated as partially shaded (PS) (Supplementary Material Figure S2B). The bioassays were carried out inside cages (15 × 60 × 75 cm) covered with fine mesh to avoid predators from interfering with the experiments. Blastospore virulence bioassays over time (persistence) were performed under PS conditions. The temperature and humidity were monitored constantly using dataloggers (Watchdog Ltd., Aurora, IL, USA).

### 2.8. Statistical Analysis

For survival data, the homogeneity of the repetitions was analyzed using the log-rank test at a 95% significance level. Homogenous results were then pooled for survival curve analysis. The average survival time (S_50_) was calculated using the Kaplan-Meier method [33]. Statistical differences between the survival curves of different treatments were compared using the log-rank test. The results for all the control groups were pooled and only one control survival curve was shown.

Comparisons of *Ae. aegypti* endpoint survival percentages for the different treatments were assessed using one-way analysis of variance (ANOVA). When significant differences were observed between treatments, the data were further analyzed by Duncan’s post-hoc test at the 5% level.

## 3. Results

### 3.1. Comparison of Blastospore and Conidial Virulence under Laboratory Conditions

**Figure 1:**
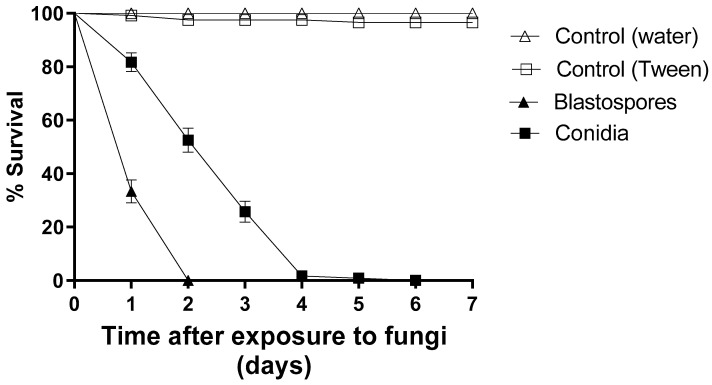
Daily survival of *Aedes aegypti* larvae exposed to *Metarhizium anisopliae* blastospores or conidia (1 × 10^7^ propagules mL^−1^) and their respective control groups (either pure distilled water or 0.01% Tween 80) under laboratory conditions. The results are shown as mean % survival with bars being standard error of the mean. Note: when bars are not shown, the SE was very low.

Blastospores caused a 100% reduction in larval survival after two days of exposure, whereas conidia reduced larval survival by approximately 50% at the same time point. Furthermore, conidia resulted in a 100% reduction of larval survival only on the sixth day of the bioassay (Figure 1). During seven days of monitoring, 100% and 97% of the larvae survived in the two control groups. The larvae exposed to blastospores had an S_50_ of 1 day and the larvae exposed to conidia an S_50_ of 3 days. The treatments using blastospores or conidia differed statistically from each other and from their respective controls [F_8.11_ = 49.976; *p* < 0.05].

### 3.2. Blastospore Persistence under Laboratory Conditions

**Figure 2:**
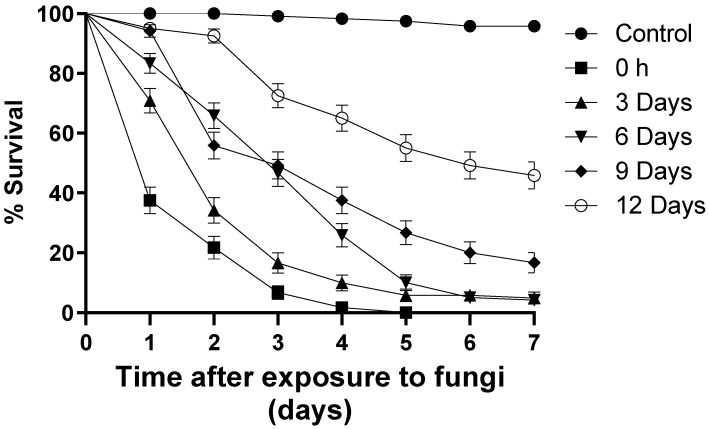
Daily survival of *Aedes aegypti* larvae exposed to *Metarhizium anisopliae* blastospores (1 × 10^7^ propagules mL^−1^) at different times following preparation of blastopores suspensions. The results are shown as mean % survival with bars being standard error of the mean. Note: when bars are not shown, the SE was very low.

Larvae exposed to blastospores at time zero (T0) reduced survival by 100% on the fifth day of monitoring (S_50_ = 1 day) (Figure 2). At the end of the bioassays, 4.1% of the larvae exposed to blastospores at T3 survived (S_50_ = 3 days), followed by T6 (4.8% survival; S_50_ = 2 days), T9 (16.6% survival; S_50_ = 3 days), and T12 (45.8% survival; S_50_ = 6 days) (Figure 2). In the control group, 95% of the larvae survived during the bioassay. Regardless of the treatment, larvae exposed to blastospores had a significant reduction [F_12.17_ = 5.071; *p* < 0.05] in survival when compared with the control group.

### 3.3. Assessment of Blastospore Virulence under Semi-Field Conditions: Totally Shaded (TS) and Partially Shaded (PS)

**Figure 3:**
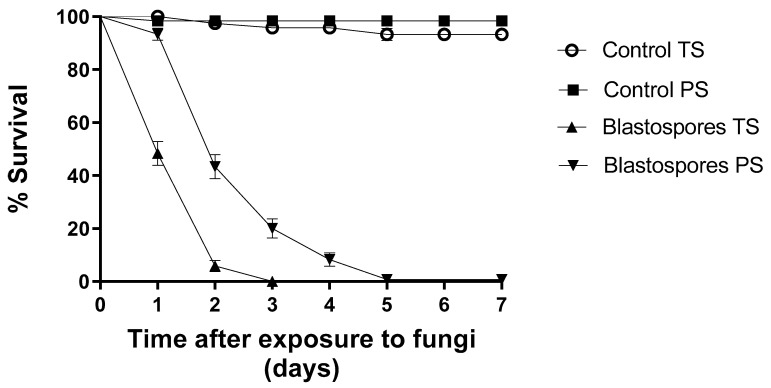
Survival curves of *Aedes aegypti* larvae following exposure to *Metarhizium anisopliae* blastospores under totally shaded (TS) and partially shaded (PS) conditions over 7 days. The blastospore concentration was 1 × 10^7^ mL^−1^. Control treatments were carried out with distilled water only. The results are shown as mean % survival with bars being standard error of the mean. Note: when bars are not shown, the SE was very low.

Under totally shaded conditions, blastospores reduced larval survival by 100% on the third day of monitoring (Figure 3). Here, the ambient temperature ranged from 25 to 28 °C, and the relative humidity (RH) ranged from 71 to 82% during the experiment. Under partially shaded conditions, blastospores reduced larval survival by 20% within three days of exposure (Figure 3); however, after seven days, larval survival decreased to 0.8%. During this experiment, the temperature ranged from 25 to 38 °C and the RH ranged from 44 to 55%. The control group survival rates for TS and PS conditions were 97% and 98%, respectively.

After 48 h of monitoring, 5.8% (S_50_ = 1 day) of the larvae survived exposure to blastopores under TS conditions (Table 1). On the other hand, blastospores under partially shaded conditions reduced larval survival by 43.3% (S_50_ = 2 days). Once again, regardless of treatment, blastospores significantly reduced larval survival [F_8.11_ = 389.200; *p* < 0.05] in comparison to the control groups (Table 1).

### 3.4. Blastospore Persistence under Semi-Field Conditions

**Figure 4:**
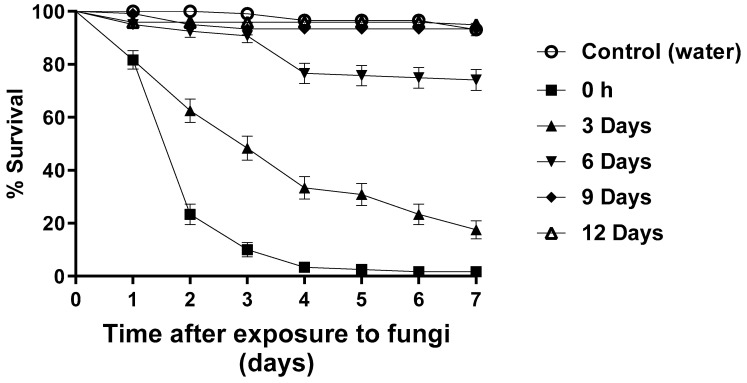
Survival curves of *Aedes aegypti* larvae exposed to *Metarhizium anisopliae* blastospores during persistence bioassays under partially shaded conditions over 7 days. The blastospore concentration was 1 × 10^7^ mL^−1^. Control treatments were carried out with distilled water only. The results are shown as mean % survival with bars being standard error of the mean. Note: when bars are not shown, the SE was very low.

The bioassays to investigate blastospore persistence were carried out under partially shaded (PS) conditions. After seven days, approximately 1% of larvae survived exposure to blastospores at T0. However, 17% of the larvae survived after being exposed to blastospores at T3, followed by 76% (T6), 93% (T9), and 95% (T12). The control group larval survival was 93% (Figure 4).

After 48 h, larval survival ranged from 23.3 to 100% (Table 2). Larvae exposed to blastospores at T0 and T3 had significantly reduced survival (*p* < 0.05) compared with T6, T9, T12, and the control group. However, the reduction in survival was not statistically different when comparing the groups exposed to blastospores at T6, T9, and T12 and the control group [F_12.17_ = 24.006; *p* < 0.05]

## 4. Discussion

Here, we investigated the virulence of *M. anisopliae* (isolate LEF 2000) blastospores and conidia against *Ae. aegypti* larvae under laboratory conditions, and blastospore virulence under semi-field conditions. This is the first time that blastospores have been tested against mosquito larvae in the field. The persistence of blastopores was also investigated under both laboratory conditions and semi-field conditions.

Blastospores were significantly more virulent than conidia to *Ae. aegypti* larvae. No larvae remained alive after a 2-day exposure to blastopores, whereas after the same time period, approximately 50% of the larvae exposed to conidia were still alive. Previously published data demonstrated that *Ae. aegypti* larvae are more susceptible to infection by blastospores than conidia under laboratory conditions [15]. According to Alkhaibari et al. [15], the higher virulence attributed to blastospores when compared to conidia is due to the presence of a thinner cell wall, which facilitates the secretion of fungal enzymes onto the insect cuticle, and also the production of large amounts of mucilage, which facilitated the adhesion of the fungus to the insect cuticle, accelerating the infection process.

Blastospores of the entomopathogenic fungus *Tolypocladium cylindrosporum* were also more virulent than conidia against *Aedes sierrensis* mosquito larvae [34]. The hydrophilic characteristics of blastospores mean that they are able to disperse in an aqueous environment. This characteristic may facilitate the interaction between the fungus and larval integument [30], different from that of conidia, which are hydrophobic and are less likely to adhere to the larval cuticle in an aquatic environment [31]. Blastospores were also more virulent than conidia to *Ae. aegypti* pupae, an important characteristic when considering that this phase of development only lasts 2–3 days [27]. Although the results confirmed the higher virulence of blastospores to *Ae. aegypti* larvae, for mosquito biological control in the field, it is important to establish the virulence of the propagules over time as well as application protocols, due to the abiotic adversities found in this environment.

Conventional control measures against larvae involve the elimination of breeding sites (plastic bags, bottles, tyres) and the application of larvicides into receptacles that cannot be eliminated, such as drains and water tanks [35]. Chemical and biological control agents need to be applied to recipients that are putative breeding sites as a preventative measure. Therefore, the control agent should have a longer active time in the field, although this could be considered a problem for some persistent or toxic chemicals, limiting their use in, for example, residential water tanks.

The virulence of blastospores was observed here over different time periods by preparing all blastospore suspensions as a single batch and then placing the larvae in these suspensions at different times post-preparation. The most virulent propagules were those to which the larvae were exposed immediately following preparation (time zero), in which case, a 100% reduction in survival was seen after 5 days. The virulence gradually fell when larvae were added to cups with blastospore suspensions at different time periods of up to 12 days, when the experiment was terminated. For larvae placed in blastospore suspensions 12 days after initial preparation, approximately 50% of the larvae remained alive 7 days later. Although larval survival for all of the blastospore treatments (0 h up to 12 days) was significantly different from the control survival percentages, the results indicated the need for further work on blastospore formulations in order to increase persistence.

Similar results were seen when testing blastospores against adult *Ae. aegypti* [26], where the virulence declined rapidly following the application of blastospores to black cloths. In that case, it was possible to increase persistence by adding vegetable oil to the blastospore suspensions. This approach is currently being tested with the aim of increasing the persistence of blastospores against larvae.

Compared to conidia, blastospores appear to have a low adaptation to the critical condition caused by abiotic stress. Some researchers have modified the fermentation process to produce blastospores that are more tolerant to stress. These types of study have shown that osmotic stress during fermentation promotes the production of more tolerant blastospores [36,37,38]. Furthermore, modifying the media by increasing the glucose concentration above 40 g/L, or encapsulating the propagule in calcium alginate with nutrients to increase viability in stressful conditions may have a positive effect [36,39,40]. Blastospore granules in soil used to control the tick *Rhipicephalus microplus* under semi-field conditions presented a decrease in fungal viability over time [14]. Fungal viability dropped significantly 15 days after the first application. The authors attributed the reduced viability to climatic factors, such as high levels of rainfall and high RH, that occurred during the tests. Nevertheless, none of these studies tested the persistence of blastospores in an aquatic environment under field conditions. Unsurprisingly, blastospores are negatively affected in terrestrial environments where they are subjected to rapid desiccation. However, the decline in virulence in an aquatic environment was unexpected. It was interesting to note that blastospores could germinate in the water and produce conidiophores and conidia on the water surface (personal observation), which could infect any remaining larvae alive in the recipients. As mentioned before, conidia are more resistant to ambient conditions because they possess a thick cell wall containing hydrophobins that protect the conidia against environmental stress [41]. The results of virulence bioassays when testing entomopathogenic fungi in the laboratory may not necessarily correlate with effectiveness against target insects in the field. This is due to the fact that the external environmental conditions are more challenging for fungal survival compared to the laboratory environment.

In the bioassays performed under totally shaded (TS) natural environmental conditions, it was demonstrated that no larvae remained alive by the third day of the experiment. In the same type of field bioassay carried out under partially shaded conditions (PS), with direct exposure to sunlight at specific times of the day, it was possible to verify that even at ambient temperatures of between 25 and 38 °C, *M. anisopliae* blastospores were still able to reduce the larval survival to almost zero over a five-day period. *Aedes aegypti* females are known to prefer oviposition sites located in shaded areas, as these sites reduce environmental stress on the immature stages [42]. Therefore, it would be unusual for larvae to develop in recipients that are in unshaded positions. The two environmental regimes chosen here were those which females would normally select as oviposition sites.

Unfortunately, blastospore virulence rapidly decreased over time when suspensions were maintained in the field before placing larvae in the recipients. Here, the persistence of blastospores was tested under PS conditions and only the 0-h and 3-day treatments resulted in significant reductions in larval survival (Figure 4 and Table 2). Although survival was slightly reduced (approx. 8%) when larvae were exposed to suspensions that had been maintained on the veranda for 6 days, these data and those for the longer time periods were not significantly different to the control survival.

Solar radiation can influence the germination capacity of the fungus and consequently its virulence [43,44,45]. UV-B radiation can permanently or temporarily block the cell cycle, preventing or considerably delaying fungal germination [46]. However, some adjuvants can improve the persistence of microbial agents in the field by protecting them from inactivation by sunlight [47]. Using entomopathogenic fungi in oil-based formulations is a promising strategy to preserve them from negative environmental effects [48]. Oil formulations prolong propagule survival and decrease sensitivity to UV radiation when compared to aqueous suspensions [49]. Solar radiation and temperature may have been factors that caused a delay in the rate of larval infection. Ultraviolet (UV) radiation is responsible for reducing the virulence of entomopathogenic fungal conidia [50].

The present study showed that under semi-field conditions, *M. anisopliae* (isolate LEF 2000) blastospores are virulent against the larvae of a natural strain of *Ae. aegypti*, and have the potential to be used in the biological control of this vector. However, further work on formulations to protect the blastospores from solar radiation needs to be carried out.

## 5. Conclusions

This study shows for the first time that blastospores are virulent against larvae of a native strain of *Ae. aegypti,* obtained from field-collected eggs. *Metarhizium anisopliae* blastospores were more virulent than conidia to *Ae. aegypti* larvae under laboratory conditions. *Metarhizium anisopliae* blastospores were also virulent to *Ae. aegypti* larvae under conditions that simulated a natural situation in the field, but virulence declined over time.

## Figures and Tables

**Table 1:** Survival (means ± SD) of *Aedes aegypti* larvae following exposure to *Metarhizium anisopliae* blastospores when maintained under totally shaded (TS) or partially shaded (PS) conditions.

Treatments	% Survival (±SD)	S_50_
Blastospores TS	5.8 ± 2.1 c	1
Blastospores PS	43.3 ± 4.5 b	2
Control TS	97.5 ± 1.3 a	ND
Control PS	98.3 ± 1.1 a	ND

Note: Survival was evaluated 48 h after exposure to the fungus under two ambient regimes. The S_50_ data was calculated on day 7 of the experiment. Results followed by the same letter indicated that there was no significant difference between means using ANOVA and Duncan’s post hoc test (5% probability). Values for S_50_ were calculated using log-rank survival analysis. ND = Not determined due to high survival values.

**Table 2:** *Aedes aegypti* survival (% mean ± SD) when larvae were placed in blastospore suspensions at different times after preparation and the cups with larvae + fungus were maintained under partially shaded (PS) conditions.

Treatments	% Survival ± SD	S_50_
0 h	23.3 ± 3.8 c	2
3 days	62.5 ± 4.4 b	3
6 days	92.5 ± 2.4 a	ND
9 days	95 ± 1.9 a	ND
12 days	95.8 ± 1.8 a	ND
Control (water)	100 a	ND

Note: Survival percentages were evaluated 48 h after placing the larvae in the cups with blastospore suspensions. The S_50_ values were calculated from data obtained after 7 days. Results followed by the same letter indicated that there was no significant difference between means using ANOVA and Duncan’s post hoc test (5% probability). Values for S_50_ were calculated using log-rank survival analysis. ND = Not determined due to high survival values.

## Data Availability

All data will be made available upon request to the corresponding author.

## Supplementary Materials

The following supporting information can be downloaded at: https://www.mdpi.com/article/10.3390/jof9070759/s1, Figure S1: Ovitrap used for collecting Aedes eggs in the field; Figure S2: Two types of field conditions used in the evaluation of blastospore virulence. (A) Totally shaded conditions and e (B) Partially shaded conditions. The large cages were used to prevent predators from interfering with the experiments. The partially shaded conditions had direct sunlight from approximately 11 am to 4 pm.
