# Supplementary material for: The Potential of Metarhizium anisopliae Blastospores to Control Aedes aegypti Larvae in the Field
Source: J Fungi (Basel). 2023 Jul 18;9(7):759. doi: 10.3390/jof9070759 (PMC10381131; doi:10.3390/jof9070759)
Supplement: Supplementary file 1 [file jof-09-00759-s001.zip › jof-2461400-supplementary.pdf]

Journal of
Fungi

MDPI

Supplementary material

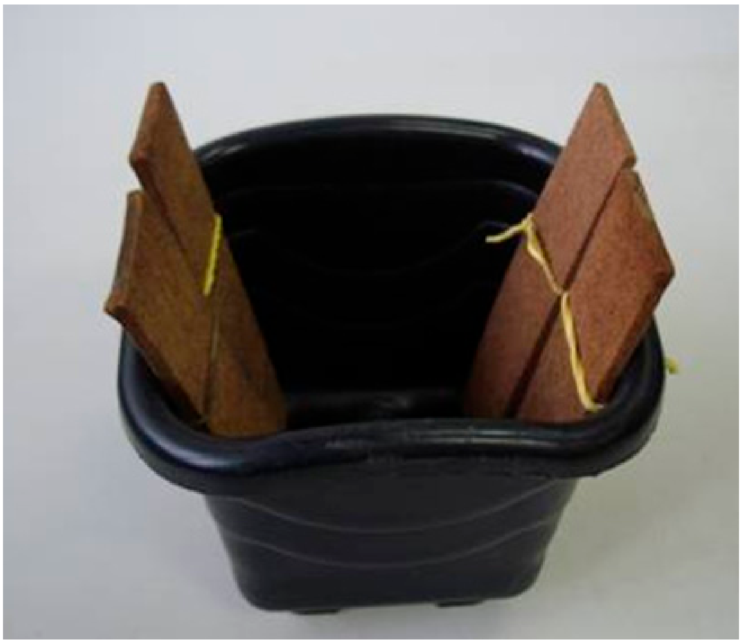

Figure S1. Ovitrap used for collecting *Aedes* eggs in the field.

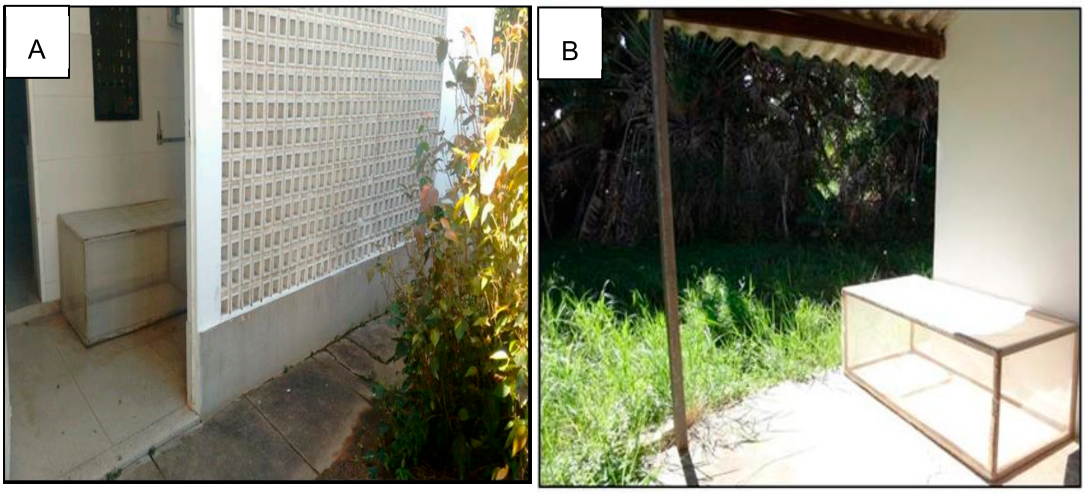

Figure S2. Two types of field conditions used in the evaluation of blastospore virulence. (**A**) Totally shaded conditions and e (**B**) Partially shaded conditions. The large cages were used to prevent predators from interfering with the experiments. The partially shaded conditions had direct sunlight from approximately 11 am to 4 pm.
